# Chromogenic anti-FXa assay calibrated with low molecular weight heparin in patients treated with rivaroxaban and apixaban: possibilities and limitations

**DOI:** 10.11613/BM.2020.010702

**Published:** 2019-12-15

**Authors:** Sandra Margetić, Ivana Ćelap, Diana Delić Brkljačić, Nikola Pavlović, Sandra Šupraha Goreta, Ivana Kobasić, Arijana Lovrenčić-Huzjan, Vanja Bašić Kes

**Affiliations:** 1 Department of Clinical Chemistry, Sestre milosrdnice University Hospital Center, Zagreb, Croatia; 2 Department of Cardiovascular Diseases, Sestre milosrdnice University Hospital Center, Zagreb, Croatia; 3 Department of Biochemistry and Molecular Biology, Faculty of Pharmacy and Biochemistry, University of Zagreb, Croatia; 4 Department of Neurology, Sestre milosrdnice University Hospital Center, Zagreb, Croatia

**Keywords:** apixaban, DOAC, factor Xa inhibitors, LMWH, rivaroxaban

## Abstract

**Introduction:**

Clinical application of rivaroxaban and apixaban does not require therapeutic monitoring. Commercial anti-activated factor X (anti-FXa) inhibition methods for all anti-FXa drugs are based on the same principle, so there are attempts to evaluate potential clinical application of heparin-calibrated anti-FXa assay as an alternative method for direct FXa inhibitors. We aimed to evaluate relationship between anti-FXa methods calibrated with low molecular weight heparin (LMWH) and with drug specific calibrators, and to determine whether commercial LMWH anti-FXa assay can be used to exclude the presence of clinically relevant concentrations of rivaroxaban and apixaban.

**Materials and methods:**

Low molecular weight heparin calibrated reagent (Siemens Healthineers, Marburg, Germany) was used for anti-FXa activity measurement. Innovance heparin (Siemens Healthineers, Marburg, Germany) calibrated with rivaroxaban and apixaban calibrators (Hyphen BioMed, Neuville-sur-Oise, France) was used for quantitative determination of FXa inhibitors.

**Results:**

Analysis showed good agreement between LMWH calibrated and rivaroxaban calibrated activity (κ = 0.76) and very good agreement with apixaban calibrated anti-Xa activity (κ = 0.82), respectively. Low molecular weight heparin anti-FXa activity cut-off values of 0.05 IU/mL and 0.1 IU/mL are suitable for excluding the presence of clinically relevant concentrations (< 30 ng/mL) of rivaroxaban and apixaban, respectively. Concentrations above 300 ng/mL exceeded upper measurement range for LMWH anti-FXa assay and cannot be determined by this method.

**Conclusion:**

Low molecular weight heparin anti-FXa assay can be used in emergency clinical conditions for ruling out the presence of clinically relevant concentrations of rivaroxaban and apixaban. However, use of LMWH anti-FXa assay is not appropriate for their quantitative determination as an interchangeable method.

## Introduction

Direct oral anticoagulants (DOACs) have been increasingly used for the prevention and treatment of thromboembolic diseases in recent years. Compared to vitamin K antagonists (VKAs), clinical application of DOACs does not require routine coagulation monitoring. However, according to the present expert opinions, there are special clinical situations in which laboratory measurement of DOACs in plasma should be performed, including bleeding or thromboembolic events (acute stroke), emergency surgical or invasive procedures, extremes of body weight, renal and/or liver failure resulting with reduced drug elimination and suspected non-compliance or overdose (*1*-*4*). There are the two main goals of quantitative determination of DOACs in above-mentioned clinical situations. First, to assess the degree of anticoagulation in certain clinical situations, and second to exclude clinically relevant drug concentrations in circulation.

Introduction of DOACs into clinical practice has faced haemostasis laboratories with new challenges. On one hand, treatment of patients with DOACs has a significant impact on the results of screening coagulation tests, *i.e.*, prothrombin time (PT) and activated partial thromboplastin time (APTT). Knowing the impact of DOACs on the results of screening coagulation tests is a precondition for the correct interpretation of these assays. However, PT and APTT are not appropriate for direct FXa inhibitors, neither for quantifying drug concentration and reliable assessment of their anticoagulant effect, nor to exclude the presence of clinically relevant drug concentrations in the circulation due to the high differences in sensitivities of individual commercial PT and APTT reagents (*5*-*9*). Therefore, the second major challenge for haemostasis laboratories refers to implementing methods that allow quantitative determination of DOACs in certain clinical situations in order to help clinical decision making (*1*, *9*-*12*). Furthermore, the need for quantitative methods for DOACs measurement is strongly supported by increasingly published frequent cases of treated patients in which extensive or fatal bleeding is described (*13*).

Therefore, in parallel with the introduction of DOACs into clinical practice, research has also been focused on the development of specific coagulation methods for quantitative determination of these drugs. In order to achieve this purpose, chromogenic assays with drug specific calibrator materials were recently introduced as promising quantitative methods for particular DOAC drug (*14*-*17*). The implementation of specific methods for quantifying DOAC concentrations in haemostasis laboratories has significantly increased recently. On the other hand, the same chromogenic anti-activated factor X (anti-FXa) principle method for both unfractioned heparin (UFH) and low molecular weight heparin (LMWH) monitoring is widely used for years. Commercial assays developed for all anti-FXa inhibitors, including heparins and related drugs, are based on the inhibitory effect of the drug present in patient´s plasma on activated factor Xa (FXa) in the test system. However, assays intended to measure particular direct or indirect anti-FXa drug need a calibration curve obtained with a drug specific calibrators. On the other hand, it has also been recently shown that assays, even calibrated with drug specific calibrators, are not fully specific, meaning that assay calibrated with specific calibrators for one drug can also measure other anti-FXa drugs (*18*). These observations resulted with first attempts for potential use of heparin calibrated anti-FXa assay as an alternative method for direct FXa inhibitors, such as rivaroxaban and apixaban (*19*-*23*).

Thus, we have hypothesized that LMWH calibrated anti-FXa assay could be used in emergency cases for detection of rivaroxaban and apixaban presence when specific method is not available. The objective of this study was to assess the relationship between LMWH calibrated anti-FXa chromogenic assay and the same chromogenic method for rivaroxaban and apixaban calibrated with drug specific calibrators. Our particular interest was to evaluate whether commercial method for measuring LMWH anti-FXa activity can be used to exclude the presence of clinically relevant concentrations of rivaroxaban and apixaban in circulation.

## Materials and methods

### Materials

The study was performed as a part of the research project IP-2016-06-8208 entitled „*New oral anticoagulants: relationship between drug concentration and anticoagulant effect*“, funded by the Croatian Science Foundation. The institutional ethic committee has approved our study protocol as a part of the mentioned research project. All patients signed written informed consent according to the ethical guidelines following the Declaration of Helsinki.

Samples from patients taking rivaroxaban and apixaban were collected from July to December 2018 at the Department of Neurology and Department of Cardiovascular Diseases, Sestre Milosrdnice University Hospital Center. A total of 61 samples from patients taking rivaroxaban (31 peak and 30 trough) and a total of 53 (30 peak and 23 trough) samples from patients taking apixaban were used in the study. Blood samples were taken from the same patients and on the same day to obtain both, trough (immediately prior the next drug dose) and peak (two hours after drug administration) concentrations of rivaroxaban and apixaban in plasma. All patients were treated with standard and equal drug doses for non-valvular atrial fibrillation (NVAF) clinical indication (*i.e.* rivaroxaban 20 mg once daily and apixaban 5 mg twice daily).

Venous blood samples for determinations of rivaroxaban and apixaban concentrations were collected in Vacuette tubes (Greiner Bio-One, Kremsmünster, Austria) containing 3.2%-trisodium citrate (volume 3.5 mL). All samples were centrifuged at room temperature for 10 minutes at 1800xg to obtain platelet poor plasma, aliquoted into labelled tubes and stored at - 20°C until analysis. Samples for the conducted study have been chosen in order to cover as much as possible the whole measurement range (up to 500 ng/mL) for both rivaroxaban and apixaban concentrations.

### Methods

All coagulation assays were performed on Behring Coagulation System XP (BCSXP) analyser (Siemens Healthineers, Marburg, Germany). Low molecular weight heparin anti-FXa activity was determined in all samples by chromogenic method using original manufacturers´ reagent kit (Berichrom heparin, Siemens Healthineers, Marburg, Germany) and LMWH calibrator (Siemens Healthineers, Marburg, Germany). Results were expressed in anti-FXa heparin equivalent international units (IU/mL). The concentrations of rivaroxaban and apixaban were measured using specific chromogenic anti-FXa assay (Innovance heparin, Siemens Healthineers, Marburg, Germany), calibrated with specific calibrators for rivaroxaban and apixaban (Hyphen BioMed, Neuville-sur-Oise, France). Concentrations of rivaroxaban and apixaban were expressed in ng/mL.

### Statistical analysis

Data distribution was tested by Komolgorov-Smirnov test. Since subgroups with peak and trough concentrations of each drug had 31 samples or less, non-parametric statistics was used. Agreement between LMWH calibrated and drug specific calibrated anti-FXa activities was tested using kappa statistics. To test the comparability between LMWH calibrated and drug specific calibrated anti-FXa activities, results of DOAC concentrations were divided in three categories. The first category included results lower than the lowest of trough concentrations, the second category included results within expected range of concentrations and the third category included results higher than the highest of peak concentrations. Expected ranges for therapeutic drug concentrations for patients with non-valvular atrial fibrillation are adopted from published guidelines by Gosselin *et al.* (*9*). Low molecular weight heparin calibrated FXa activity was categorized in three categories according to measurement range (0.05 – 1.26 IU/mL). Results of DOACs concentrations and LMWH anti-FXa activity are presented as median with 95% confidence interval (95% CI) and interquartile range (IQR). Differences between peak and trough concentrations of DOACs and LMWH anti-FXa activities were tested using the nonparametric Mann-Whitney test.

Receiver-operating characteristics (ROC) analysis was done to determine cut-off values of LMWH calibrated anti-FXa activity which corresponds to rivaroxaban and apixaban values < 30 ng/mL and < 50 ng/mL. Those cut-off values were used as suggested for the treatment of patients with excessive bleeding and perioperative management by Levy *et al.* (*24*). P value < 0.05 was considered statistically significant. Statistical analysis was performed using MedCalc for Windows, version 19.0.3 (MedCalc Software, Ostend, Belgium). Correlation scatter analysis was performed using Microsoft Excel version 2010 (Microsoft Corporation, Redmond Washington).

## Results

The rivaroxaban concentrations obtained by chromogenic anti-FXa method with drug specific calibrators ranged from 62 to 433 ng/mL for peak and from 4 to 83 ng/mL for trough concentrations. The apixaban peak and trough concentrations ranged from 73 to 415 and from 13 to 98 ng/mL, respectively.

Results of rivaroxaban and apixaban peak and trough concentrations as well as LMWH anti-FXa activites are presented in Table 1. Differences between rivaroxaban and apixaban peak concentrations were not statistically significant (P = 0.745). On the contrary, trough concentrations of apixaban were significantly higher than rivaroxaban trough concentrations (P < 0.001). Considering appropriate LMWH anti-FXa activities for both peak and trough concentrations, the results demonstrated significant difference between LMWH anti-FXa activities for rivaroxaban and apixaban peak concentrations (P = 0.011), whereas the difference was not statistically significant for LMWH anti-FXa activities measured in samples with trough concentrations of both drugs (P = 0.099).

**Table 1:** Peak and trough concentrations of rivaroxaban and apixaban determined with drug specific calibrators and LMWH-calibrated anti-FXa activities

**Drug**	**N**	**Concentration with drug specific calibrators, ng/mL**	P*	**LMWH-calibrated anti-FXa activity, IU/mL**	**P^†^**
**Apixaban peak**	30	157.0 (122.0 – 200.8); 119.0 – 224.0	0.745	0.80 (0.63 – 1. 00); 0.68 – 0.95	0.011
**Rivaroxaban peak**	30	150.0 (126.5 – 181.8); 122.0 – 200.0		0.59 (0.46 – 0.84); 0.52 – 0.72	
**Apixaban trough**	23	48.0 (42.3 – 59.5); 44.0 – 57.7	< 0.001	0.14 (0.11 – 0.27), 0.11 -0.26	0.099
**Rivaroxaban trough**	31	19.7 (11.0 – 42.0); 12.2 – 37.0		0.11 (0.00 – 0.26); 0.00 – 0.23	
Concentrations are presented as median (95% confidence interval) and interquartile range. LMWH - low molecular weight heparin. P* between apixaban and rivaroxaban peak, and apixaban and rivaroxaban trough concentrations measured using drug specific calibrators. P^†^ between apixaban and rivaroxaban peak and apixaban and rivaroxaban trough concentrations measured using LMWH-calibrated anti-FXa activity assay. P < 0.05 was considered statistically significant.					

Kappa statistics showed good agreement between LMWH calibrated and rivaroxaban calibrated FXa activities (κ = 0.76) (Table 2). Further, agreement between LMWH calibrated and apixaban calibrated FXa activities has been shown as a very good (κ = 0.82) (Table 3).

**Table 2:** Agreement between categories of rivaroxaban determined with drug specific calibrators and with LMWH-calibrated anti-FXa activity assay

		**Rivaroxaban (ng/mL)**			
		< 12	12 - 343	> 343	Total
**LMWH** **(IU/mL)**	< 0.05	8	4	0	12
	0.05 – 1.26	0	46	0	46
	> 1.26	0	1	2	3
**Total**		8	51	2	61
LMWH – low molecular weight heparin. Rivaroxaban concentrations were categorized according to expected therapeutic ranges for apixaban (12 - 343 ng/mL) given by Gosselin *et al.* (*19*). LMWH categories were assigned according to the measurement range (0.05 –1.26 IU/mL) of the LMWH anti-FXa assay used in our study. Kappa (κ) = 0.76 (95%CI 0.56 – 0.96).					

**Table 3:** Agreement between categories of apixaban determined with drug specific calibrators and with LMWH-calibrated anti-FXa activity assay

		**Apixaban (ng/mL)**			
		< 41	41 - 321	> 321	Total
**LMWH** **(IU/mL)**	< 0.05	3	0	0	3
	0.05 – 1.26	2	46	0	48
	> 1.26	0	0	2	2
**Total**		5	46	2	53
LMWH – low molecular weight heparin. Apixaban concentrations were categorized according to expected therapeutic ranges for apixaban (41 – 321 ng/mL) given by Gosselin *et al.* (*19*). LMWH categories were assigned according to the measurement range (0.05 – 1.26 IU/mL) of the LMWH anti-FXa assay used in our study. Kappa (κ) = 0.82 (95%CI 0.57 – 1.00).					

As presented in Table 4, the results of ROC analysis have shown cut-off value at 0.05 IU/mL of LMWH anti-FXa activity with 100% (95%CI 83.2 – 100) sensitivity and 100% negative predictive value for apixaban concentrations < 30 ng/mL. Further, cut-off at 0.10 IU/mL of LMWH anti-FXa activity with 100% (95%CI 76.8 – 100) and 100% negative predictive value was shown for rivaroxaban concentrations < 30 ng/mL.

**Table 4:** Cut-off values of LMWH calibrated anti-FXa activity for rivaroxaban and apixaban arbitrary defined concentrations

**Drug**	**Cut-off** **LMWH anti-FXa activity (IU/mL)**	**AUC** **(95%CI)**	**P**	**Sensitivity, %** **(95%CI)**	**Specificity, %** **(95%CI)**	**PPV** **(95%CI)**	**NPV** **(95%CI)**
**Apixaban** < 30 ng/mL*	> 0.05	1.00 (0.85 – 1.00)	< 0.001	100 (83.2 – 100.0)	100 (29.2 – 100.0)	100 (NA)	100 (NA)
**Rivaroxaban** < 30 ng/mL*	> 0.10	0.99 (0.88 – 1.00)	< 0.001	100 (76.8 – 100.0)	93.8 (69.8 – 99.8)	93.3 (67.7 – 98.9)	100 (NA)
**Apixaban** **< 50 ng/mL^†^**	> 0.12	0.98 (0.81 – 1.00)	< 0.001	100 (66.4 – 100.0)	78.6 (49.2 – 95.3)	75 (52.4 – 89.1)	100 (NA)
**Rivaroxaban** **< 50 ng/mL^†^**	> 0.23	0.89 (0.73 – 0.98)	< 0.001	100 (39.8 – 100.0)	84.6 (65.1 – 95.6)	50 (28.9 – 71.1)	100 (NA)
AUC – area under curve. PPV - positive predictive value. NPV - negative predictive value. NA – not applicable. LMWH – low molecular weight heparin. *Arbitrary defined cut-off value related to perioperative reversal drug administration (*24*). ^†^Arbitrary defined cut-off value related to reversal drug administration in bleeding patients (*24*).							

## Discussion

In this study, we evaluated relationship between two chromogenic anti-FXa methods, one calibrated with LMWH and the other with drug specific calibrators for rivaroxaban and apixaban. The main two questions that we wanted to answer were whether LMWH-calibrated anti-FXa activity assay can be used: 1) for excluding clinically relevant concentrations of direct anti-FXa drugs in circulation and 2) for quantifying rivaroxaban or apixaban concentrations in plasma as an alternative method to the chromogenic assays calibrated with specific drug.

Our study has shown good agreement between LMWH anti-FXa activity and concentrations of rivaroxaban. Furthermore, a very good agreement between LMWH anti-FXa activity and apixaban concentrations has been shown. A cut-off value of 0.05 IU/mL corresponded to the apixaban concentration below 30 ng/mL and could be used in our laboratory for excluding the presence of apixaban with negative predictive value of 100%. In case of rivaroxaban, for concentrations < 30 ng/mL, cut-off value at 0.1 IU/mL of LMWH anti-FXa activity with negative predictive value of 100% has been obtained. We have chosen cut-off values with the highest sensitivity and negative predictive value to ensure reliable rule out method in our laboratory. Thus, our results confirmed that if quantitative chromogenic methods calibrated with drug-specific calibrators are not available, LMWH-calibrated anti-FXa activity assay could be used as an alternative method for excluding the presence of clinically relevant concentration of anti-FXa drugs with the negative predictive value of 100%. Namely, an important issue for possible clinical application of LMWH calibrated anti-FXa activity assay in excluding clinically relevant concentration of anti-FXa drugs lies in fact that some emergencies require administration of the reversal agent. The Scientific and Standardization Committee (SSC) of the International Society on Thrombosis and Hemostasis (ISTH) recommended administration of the reversal agent in the perioperative setting, if plasma concentration of direct FXa inhibitor is above 30 ng/mL, in order to ensure adequate haemostasis (*24*). In bleeding patients, antidote administration is supported at plasma concentrations above 50 ng/mL (*19*). Our study also confirmed results of Sabor *et al*. who reported that anti-FXa response is not the same at the concentrations of 30 and 50 ng/mL of rivaroxaban and apixaban (*20*).

For both rivaroxaban and apixaban, most of the values fell within the measurement range of the LMWH anti-FXa activity. Further, 3/61 and 2/53 samples with high rivaroxaban and apixaban concentrations exceeded the upper level of measurement range for LMWH calibrated anti-FXa method. When considering the upper level of the measurement range for different commercial LMWH-anti FXa activity assays, it is important to note that it is determined by the activity of the highest calibrator. In case of LMWH anti-FXa activity assay used in this study, the upper measurement range of 1.26 IU/mL corresponds to the concentrations of both rivaroxaban and apixaban of approximately 300 ng/mL. Thus, concentrations above 300 ng/mL for both drugs cannot be quantified by this test.

Our results are in accordance with those obtained by Gosselin *et al.* who have also shown that although LMWH-calibrated anti-FXa activity assay can be used for ruling out the presence of direct anti-FXa inhibitors in circulation, it is not suitable for quantitative determinations of anti-FXa drugs in plasma (*19*).

Beside of limited range of quantification of the direct anti-FXa inhibitors using LMWH-calibrated anti-FXa assays, several other issues complicate its use in the accurate assessment of anti-FXa inhibitors. First, the result for LMWH anti-FXa assay activity is expressed in international anti-FXa units/mL (anti-FXa IU/mL), whereas recommended measurement unit for direct FXa inhibitors is ng/mL. Further, there is an evidence of substantial variability between different commercial kits and calibrators for LMWH-calibrated anti-FXa assays (*19*, *21*). This fact additionally complicates comparisons between drug concentrations and anti-FXa activity reporting. Both lower and upper limits of quantification were shown to be variable and significantly limited by the calibrator measurement range. Based on these results the authors concluded that “on therapy” concentrations of rivaroxaban cannot be quantitated using UFH-, LMWH- or hybrid-calibrated anti-FXa activity assays unless significant modifications are made to extend the current reportable range of the assay. Thus, it seems that results obtained with different commercial LMWH-calibrated anti-FXa assays cannot be extrapolated between different assays. Additionally, the authors cautioned that a LMWH-calibrated anti-FXa activity might not represent the same degree of anticoagulation for apixaban as it does for LMWH (*19*).

In the light of finding possible solutions to the problems related to need for different drug specific calibrators, controls and measurement units (IU/mL and ng/mL) used in anti-FXa assays intended for different anti-FXa drugs, a group of authors have recently proposed a new concept that could contribute in solving this problem. These authors suggested a concept for a single anti-FXa based laboratory assay for all drugs that directly or indirectly inhibit FXa. The initiators of the idea of so called „Da-Xa inhibition assay“ suggest a new test that would report inhibitory activity rather than drug concentration or IU/mL (*18*). The authors assume that the strategy of universal Da-Xa inhibition assay would eliminate the need for drug specific calibrations for anti-FXa drugs, heparins and heparin derived drugs (*18*). However, with assays available at this moment related to the measurement of anti-FXa inhibitors, and based on the results of our own study, our opinion is that LMWH-calibrated anti-FXa activity assay could be applied as an informative method for the purpose of excluding or confirming clinically relevant concentrations of anti-FXa drugs in circulation. However, as this method is associated with a limited measurement range, it could not be recommended for quantifying direct anti-FXa inhibitors. Instead, a chromogenic anti-FXa assay calibrated with drug-specific calibrator materials should be used in clinical situations intended for quantitative determinations of anti-FXa drugs.

The strength of this study relies on the fact that the results are obtained in plasma of patients treated with both direct anti-FXa inhibitors, rivaroxaban and apixaban, unlike several previous studies that reported results of drug concentrations in plasma samples where the certain concentration of DOACs was added *in vitro*. Those results are significantly different from the values in the plasma of patients treated with DOACs (*25*, *26*). The benefit of our study relies in fact that wide range of concentrations of both direct anti-FXa inhibitors were evaluated, thus allowing reliable conclusions based on the study results and related to the issues that were in our focus.

The limitation of our study is that it was restricted to NVAF as the only clinical indication included. However, this clinical condition still represents the most common indication for introduction of DOACs in patient management, thus being the real representative patient population for the purpose of this research. Furthermore, a possible limitation could be the fact that we compared only one LMWH-anti-FXa assay with one drug-calibrated anti-FXa assay for rivaroxaban and apixaban available in the market. However, our intention was to compare the two methods that are in use at our laboratory in order to apply results in management of patients treated at our institution.

In conclusion, the findings of this study will improve the understanding in terms of both possibilities and limitations of LMWH-calibrated chromogenic anti-FXa assays in patients treated with direct anti-FXa inhibitors. Our results strongly suggest that LMWH calibrated anti-FXa assay has a potential as an alternative first line method for excluding the presence of significant levels of rivaroxaban and apixaban if laboratory has no available specific chromogenic anti-FXa assay calibrated with particular drug. However, in case of positive result suggesting the presence of an oral anti-FXa inhibitor in plasma, specific chromogenic assay for particular anti-FXa drug should be performed for quantitative determination of these drugs. The use of LMWH calibrated anti-FXa assay to quantify rivaroxaban and apixaban concentrations could not be recommended in routine clinical practice as the only method for quantification of anti-FXa medications. It is of crucial importance that both laboratory experts and clinicians who treat patients substantially understand the opportunities and limitations of heparin-calibrated anti-FXa assays in patients treated with anti-FXa inhibitors.
